# Epidemiological and clinical insights from SARS-CoV-2 RT-PCR crossing threshold values, France, January to November 2020

**DOI:** 10.2807/1560-7917.ES.2022.27.6.2100406

**Published:** 2022-02-10

**Authors:** Samuel Alizon, Christian Selinger, Mircea T Sofonea, Stéphanie Haim-Boukobza, Jean-Marc Giannoli, Laetitia Ninove, Sylvie Pillet, Vincent Thibault, Alexis de Rougemont, Camille Tumiotto, Morgane Solis, Robin Stephan, Céline Bressollette-Bodin, Maud Salmona, Anne-Sophie L’Honneur, Sylvie Behillil, Caroline Lefeuvre, Julia Dina, Sébastien Hantz, Cédric Hartard, David Veyer, Héloïse M Delagrèverie, Slim Fourati, Benoît Visseaux, Cécile Henquell, Bruno Lina, Vincent Foulongne, Sonia Burrel, on behalf of the SFM COVID-19 study group

**Affiliations:** 1 MIVEGEC, CNRS, IRD, Université de Montpellier, France; 2 Center for Interdisciplinary Research in Biology (CIRB), College de France, CNRS, INSERM, Université PSL, Paris, France; 3 Laboratoire CERBA, Saint-Ouen-L’Aumône, France; 4 BIOGROUP, Scientific Direction, Lyon, France; 5 Unité des Virus Émergents (UVE: Aix-Marseille Univ-IRD 190-Inserm 1207-IHU Méditerranée Infection), Marseille, France; 6 Laboratoire des agents infectieux et d’hygiène, CHU de Saint-Etienne, France; 7 CIRI, Centre International de Recherche en Infectiologie, GIMAP team, University of Lyon, University of Saint-Etienne, INSERM, U1111, CNRS UMR5308, ENS de Lyon, UCBL, Lyon, France; 8 Laboratoire de Virologie, CHU Rennes, Rennes, France; 9 Laboratory of Virology-Serology, University Hospital of Dijon Bourgogne, Dijon, France; 10 UMR PAM A 02.102 Procédés Alimentaires et Microbiologiques, Université de Bourgogne Franche-Comté/AgroSup Dijon, Dijon, France; 11 University of Bordeaux, CNRS-UMR 5234, CHU Bordeaux, Virology Department, Bordeaux, France; 12 CHU de Strasbourg, Laboratoire de Virologie, Strasbourg, France, Université de Strasbourg, INSERM, IRM UMR_S 1109, Strasbourg, France; 13 Laboratoire de Microbiologie, CHU Nîmes, Nîmes, France; 14 CHU Nantes, Nantes Université, Service de Virologie, Nantes, France; 15 Laboratoire de Virologie, Hôpital Saint Louis, APHP, INSERM U976, équipe INSIGHT, Université de Paris, Paris, France; 16 Assistance Publique-Hôpitaux de Paris, Hôpital Cochin, Service de Virologie, Paris, France; 17 National Reference Center for Respiratory Viruses, Molecular Genetics of RNA Viruses, UMR 3569 CNRS, University of Paris, Institut Pasteur, Paris, France; 18 Département de Biologie des Agents Infectieux, Laboratoire de Virologie, CHU d’Angers, Angers, France; 19 Laboratoire HIFIH, UPRES EA 3859, Université d’Angers, Angers, France; 20 Laboratoire de Virologie, CHU de Caen, UNICAEN, INSERM U1311 DYNAMICURE, Université de Caen Normandie, Caen, France; 21 CHU Limoges, Laboratoire de Bactériologie-Virologie-Hygiène, Limoges, France; 22 RESINFIT, U 1092, University of Limoges, Limoges, France; 23 Laboratoire de Virologie, CHRU de Nancy Brabois, Vandoeuvre-lès-Nancy, France; Université de Lorraine, CNRS, LCPME, Nancy, France; 24 Laboratoire de Virologie, Service de Microbiologie, hôpital européen Georges Pompidou, Assistance Publique-Hôpitaux de Paris et Unité de Génomique Fonctionnelle des Tumeurs Solides, Centre de Recherche des Cordeliers, INSERM, Université Paris, Paris, France.; 25 AP-HP, Hôpital Avicenne, Laboratoire de microbiologie clinique, Bobigny, France; 26 Henri Mondor Hospital, virology department, Créteil, France; 27 Université de Paris, Inserm, UMR 1137 IAME et Laboratoire de Virologie, Hôpital Bichat Claude Bernard, AP-HP, Paris, France; 28 Service de Virologie médicale, 3IHP, CHU Clermont-Ferrand, Clermont-Ferrand, France; 29 CNR des virus des infections respiratoires (dont la Grippe), Institut des Agents Infectieux, Hopital de la Croix Rousse, HCL, Lyon, France; 30 Pathogenesis and control of chronic and emerging infections, Université de Montpellier, UMR 1058, CHU de Montpellier, Inserm, Université des Antilles, Montpellier, France; 31 Sorbonne Université, INSERM U1136, Institut Pierre Louis d’Epidémiologie et de Santé Publique (IPLESP), AP-HP, Hôpital Pitié-Salpêtrière, Service de Virologie, Paris, France; 32 French Society of Microbiology (SFM), https://www.sfm-microbiologie.org

**Keywords:** COVID-19, SARS-CoV-2, RT-PCR, virus load, epidemiology, statistical modelling

## Abstract

**Background:**

The COVID-19 pandemic has led to an unprecedented daily use of RT-PCR tests. These tests are interpreted qualitatively for diagnosis, and the relevance of the test result intensity, i.e. the number of quantification cycles (Cq), is debated because of strong potential biases.

**Aim:**

We explored the possibility to use Cq values from SARS-CoV-2 screening tests to better understand the spread of an epidemic and to better understand the biology of the infection.

**Methods:**

We used linear regression models to analyse a large database of 793,479 Cq values from tests performed on more than 2 million samples between 21 January and 30 November 2020, i.e. the first two pandemic waves. We performed time series analysis using autoregressive integrated moving average (ARIMA) models to estimate whether Cq data information improves short-term predictions of epidemiological dynamics.

**Results:**

Although we found that the Cq values varied depending on the testing laboratory or the assay used, we detected strong significant trends associated with patient age, number of days after symptoms onset or the state of the epidemic (the temporal reproduction number) at the time of the test. Furthermore, knowing the quartiles of the Cq distribution greatly reduced the error in predicting the temporal reproduction number of the COVID-19 epidemic.

**Conclusion:**

Our results suggest that Cq values of screening tests performed in the general population generate testable hypotheses and help improve short-term predictions for epidemic surveillance.

## Introduction

Molecular testing is a key component of screening policies to control the spread of infectious diseases, and the coronavirus disease (COVID-19) pandemic has led to an unprecedented testing rate using reverse transcription PCR (RT-PCR) assays [1]. In clinical and public health practice, RT-PCR results are qualitative for viral respiratory disease diagnostics, with reports such as ‘positive’, ‘negative’, ‘uninterpretable’ and, sometimes, ‘weakly positive’. These are based on the cycles threshold, also referred to as crossing point or crossing threshold (here denoted quantification cycles (Cq)), which corresponds to the number of PCR amplification cycles required for the fluorescent signal to rise above a positive threshold. In theory, the more abundant the genetic target in the sample, the fewer the amplification cycles required to detect it. This is why numerous studies on severe acute respiratory syndrome coronavirus 2 (SARS-CoV-2) rely on Cq values to assess transmissibility [2] or study infection kinetics [3]. However, many practical and biological limitations make Cq values a poor reflector of virus load [4]. 

Few studies analyse Cq values at a population level. One explanation is that these are known to suffer from several, potentially strong, biases. Firstly, sample type and sampling quality directly affect the amount of genetic material available. Secondly, the choice of RT-PCR assay matters. Even the quality of the reagents may have a considerable effect on the number of amplification cycles required to achieve the same level of fluorescence for the same amount of target genetic material. Combining data from different laboratories helps control for these sources of variation in statistical analyses. Furthermore, the larger the dataset, the more we can detect small statistical trends even after having controlled for non-informative variables.

Here, we present a cross-sectional analysis of SARS-CoV-2 RT-PCR tests performed on samples from 2,220,212 individuals in France during the COVID-19 pandemic between 21 January and 30 November 2020 (Supplementary Figure S2 shows the daily number of tests and the number of tests per French department). Our aim was to determine firstly, if this analysis at the population level can identify cofactors of interests (e.g. age, sex, type of sample) and, therefore, new virological or immunological hypotheses and, secondly, if it can improve our ability to anticipate epidemic trends.

## Methods

We studied SARS-CoV-2 RT-PCR tests performed in 2020 in France on samples from individuals aged between 1 and 89 years. The national French database for SARS-CoV-2 RT-PCR tests (SI-DEP) collects qualitative results but Cq values are not reported. In order to focus on Cq values, this study relied on the French Society of Microbiology (SFM) network of hospital-based and private laboratories. Nationwide, databases from 21 laboratories, listed in the Supplement, were included on a voluntary basis. The geographical coverage of the tests is shown in Supplementary Figure S2B. The context in which these tests were performed varied over time. Until at least April 2020, the testing capacity was limited in the country and the majority of tests were performed on symptomatic individuals, especially in hospital settings. After May 2020, testing was more accessible and data then included screening tests performed in the general population. This change in testing context coincided with a shift in terms of screening facilities, with the majority of the tests being performed in hospital virology departments until April 2020 and in private laboratories after that. However, we do not expect that this shift led to a change in testing practice across French regions. We did not include tests for which key variables such as patient age, patient sex, laboratory geographical department, qualitative result or RT-PCR assay used were unknown. Note that one test could provide more than one Cq value if it contained probes targeting multiple viral genes. According to the national guidelines [5], it is recommended to focus on the most sensitive target to categorise levels of viral excretion. After removing the 388 Cq values that we deemed marginal and potentially unrealistic because they were smaller than 10 or larger than 45, the 95% confidence interval (CI) of the remaining values was 16.89–35.56 (details on the characteristics of the whole dataset and its variables of interest are included in Supplementary Table S1). The median and upper bound of the 95% CI were unaffected by the removal of these values and the lower bound increased marginally from 16.87 to 16.89. Overall, we were left with 793,479 tests from the same number of individuals, i.e. 35.7% of the whole database. The whole database contains tests with both negative and positive clinical results. However, we only kept tests with a Cq value and the former were less represented in the final database since samples that test negative usually do not have any reported Cq value (laboratories rarely record Cq values greater than 40).

We used a linear regression model to explore how Cq values can be explained by the following variables: patient age and sex, the number of days since the onset of symptoms (if known), the clinical sampling site (if known), the sampling facility (if known), the RT-PCR assay used, the target gene, the test’s qualitative result, the sampling date, the temporal reproduction number (*Rt*) of the epidemic on the sampling date, and a control variable. The latter corresponds to the last digit of the patient anonymity number and is expected to be independent of the Cq value. Therefore, the lowest p value associated with the control variable, which we expect to be pure noise, can be used to set the significance threshold for the other variables. We also included in the model an interaction term between sampling date and *Rt*. For this analysis, we excluded Cq values from internal controls. Univariate analyses are extremely sensitive to heterogeneity in the dataset. For instance, the age distribution from patients sampled in aged care homes is different from that in city screening facilities, and analysing the ‘sampling facility’ factor alone could yield misleading results. This is why the analysis used here was multivariate and considered all the factors listed above simultaneously. In particular, it allowed us to control for variations in the way the data were collected, e.g. the intensity or the context of the sampling.

To control for the consistency of the results for some of the factors, especially those related to the infection (e.g. the number of days since symptoms onset), we also performed the analyses only on the tests that were reported as 'positive' or 'weakly positive' (i.e. we ignored the tests labelled as negative by clinical virologists). These are shown in Supplementary Table S3.

The *Rt*, which can be interpreted as the number of secondary infections caused by a person infected at a given date *t*, was estimated using national hospital admission data and the EpiEstim method [6,7]. Furthermore, the time series analysis to explore the added value of Cq data was performed using autoregressive integrated moving average (ARIMA) models. Further details about the methods can be found in the Supplementary Methods.

## Ethical statement

This study was approved by the Institutional Review Board of the Centre Hospitalier Universitaire de Montpellier. It is registered at ClinicalTrials.gov under the identifier NCT04738331.

## Results

### Factors associated with Cq values

The adjusted R-square of the linear model was 38.8%, meaning that the factors we chose explained one-third of the variance in Cq values. The model residuals were normally distributed (Supplementary Figure S3A). Care should be taken in the interpretation given that the data were unbalanced, which is why we performed an analysis of variance (ANOVA) with type II sums of squares. All factors except the *Rt* were significantly associated with Cq values using a classical 5% p value criterion. Even for the control variable, the p value was 0.013, and patients with final digits 1 and 3 in their identification number had Cq values slightly lower (−0.19 and −0.17 cycles) than patients with a 0 as the final digit. Therefore, we set our significance thresholds to 5% of that of the control variables, i.e. 6.5 × 10^−4^, to analyse the main effects (Table). Detailed outputs of the linear model are shown in Supplementary Table S2.

**Table:** Main factors affecting Cq values of SARS-CoV-2 RT-PCR in the multivariate linear model, France, January –November 2020 (n = 793,479)

Factor	Value	Coefficient	2.5% CI	97.5% CI
Intercept		19.1	12.9	25.4
Assay	PerkinElmer	Reference		
	Genefinder	12.1	10.3	13.9
Laboratory	Lab_1	Reference		
	Lab_122	5.42	3.79	7.05
	Lab_96	−4.8	−6.71	−2.90
Result	Positive	Reference		
	Weakly positive	11.3	11.1	11.5
	Negative	16.9	16.6	17.2
Days post symptom onset	Less than 4	Reference		
	4 to 7	2.76	2.66	2.86
	8 to 14	4.90	4.73	5.08
	More than 14	5.73	5.43	6.03
Sample	Nasopharyngeal	Reference		
	Other	−1.81	−2.49	−1.14
Age	Per 20 years older	−0.541	−0.585	−0.497
Target gene	N	Reference		
	ORF1	1.03	0.949	1.12
	S	1.19	0.948	1.43
Date	Per 71 days later	−0.797	−0.903	−0.691

CI: confidence interval; Cq: quantification cycle; ORF: open reading frame; SARS-CoV-2: severe acute respiratory syndrome coronavirus 2.

We only list factors with significant effects with a 10^−3^ p value criterion. Coefficients reflect differences in Cq. For qualitative factors, the reference value is shown. See the Supplement for details about the model and the scaling of the quantitative variables.

The intercept of the linear model indicates the average Cq value for a positive test performed with the reference assay, with all factors being set to their reference value. Its magnitude (19.1 cycles) is in line with clinical practice. The importance of the noise in the dataset is illustrated by the strong effect of the testing laboratory, as well as the RT-PCR assay used (Supplementary Figure S1 provides the distributions of Cq values as a function of the assay used and its target gene).

**Figure 1:**
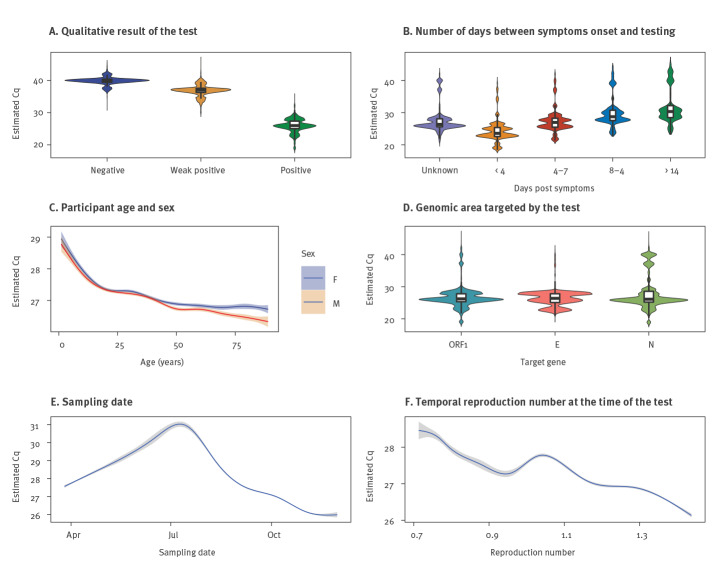
Correlations between key factors and observed Cq variations, SARS-CoV-2 RT-PCR tests, France, January–November 2020 (n = 793,479)

Despite this high level of noise, we detected a strong effect of the clinical qualitative result ('negative', 'positive' or 'weak positive') communicated by the testing laboratory (Figure 1A), with Cq differences that were even larger than those from the laboratory effect. We also found a significant difference of −1.81 cycles between the most common type of samples (nasopharyngeal) and other clinical sampling sites (mostly lower respiratory tract, but also faeces or saliva). 

Cq: quantification cycle; F: female; M: male; ORF: open reading frame; SARS-CoV-2: severe acute respiratory syndrome coronavirus 2.

For panels A, B and D, the violin plots indicate the distributions and the box plots show the 0.025, 0.25, 0.5, 0.75 and 0.975 quantiles. Panels C, E and F are obtained with a ‘loess’ smoothing model and the grey area shows the 95% confidence interval from the underlying model. The Cq values shown are not the raw values but those estimated using a multiparametric linear model to correct for biases (see Supplementary Methods for details regarding the computation of the estimated values).

The effect associated with the number of days since symptom onset was particularly strong. The number of days between symptoms onset and testing dates was known for 8.5% of the participants; their Cq values increased gradually over the reported range of days with a maximum difference of 5.73 cycles (Figure 1B). The effect was similar when removing from the analysis the tests clinically considered to be 'negative' (see Supplementary Table S3 for these sensitivity analyses).

The effect of sex had the same order of magnitude as that of the control variable and could therefore be treated as non-significant. Conversely, the factor age had a strong effect, with a decrease of 0.541 cycles per 20.1 years of age increased (Figure 1C).

The target gene of the RT-PCR assay used also yielded a significant effect. The Cq values obtained when using a probe targeting the ORF1 and S regions of the virus genome were significantly higher than when using the N gene, which was the genomic region of reference in the model (Figure 1D). 

Finally, we found that Cq values decreased with time (−0.797 cycle per day), but this effect was nonlinear (Figure 1E). This could be due to the strong variation in testing efforts in France (Supplementary Figure S2A shows that the number of tests performed varied strongly during the year), but also to variations in the epidemic trend. Indeed, although the *Rt* (inferred from hospitalisation data) on the date of sampling was not significantly associated with the Cq value according to our threshold (6.5 × 10^−4^, i.e. 5% of the lowest p value of the control variables), the interaction between the sampling date and *Rt* was nearly significant with a p value of 10^−3^ (Figure 1F), suggesting that a temporal analysis could yield additional insights.

### Anticipating epidemic spread using Cq values

The existence of a correlation between the *Rt* and the Cq values of the tests performed in a population is consistent with population dynamics theory, which predicts that in an expanding population of infected individuals, the ‘age’ of the infections, i.e. the number of days post infection, is skewed towards lower values [8]. Since Cq values have been reported to increase over the course of an infection [3], which we confirm with this analysis (Figure 1D), it has been suggested that these values could be used as an early signal to predict *Rt* [9].

**Figure 2:**
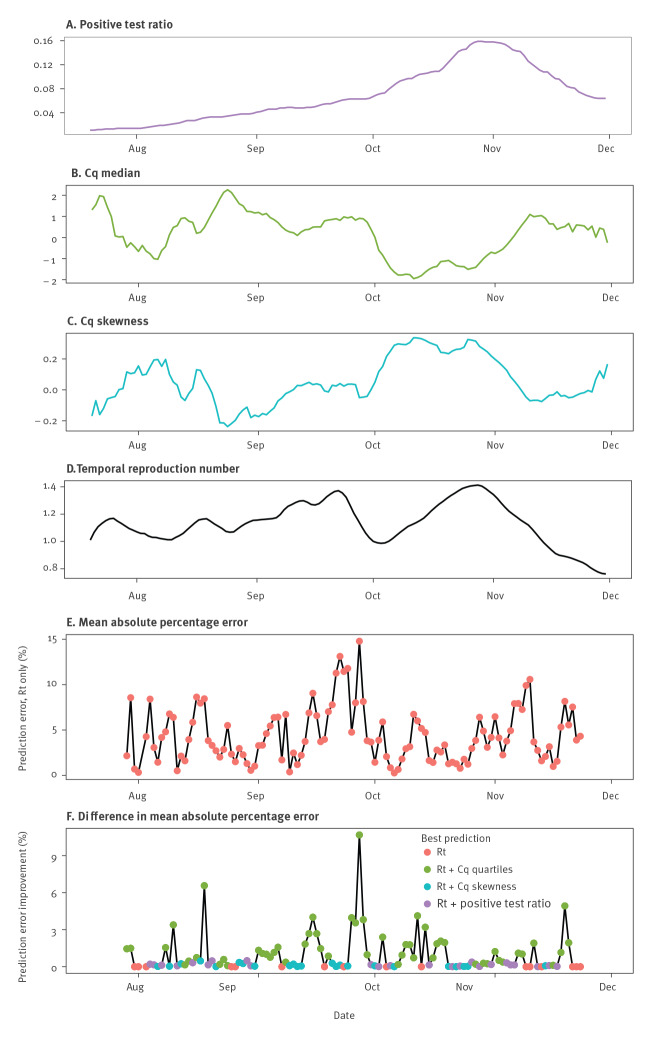
Predicting temporal reproduction number from time series related to SARS-CoV-2 RT-PCR tests, France, January–November 2020 (n = 330,611)

To investigate this question, we focused on screening data collected in the general population only from individuals aged 5 to 80 years because younger or older individuals may be associated with specific epidemiological clusters, e.g. in daycare facilities or nursing homes. We estimated the median and skewness values of the daily distribution of the Cq values. To correct for potential confounding factors, these were adjusted using a linear model (see details in the Supplementary Methods). We analysed the temporal correlation between the time series with a 7-day rolling average of this median, skew and *Rt* (Figure 2). For the median Cq value, we found a significant correlation with *Rt* that was maximised for a delay of 6–7 days (Supplementary Figure S4 provides additional information on the cross-correlation functions between *Rt* and the median or the skewness of the Cq distribution). This is consistent with *Rt* being calculated using data from hospital admissions for COVID-19, which occur at a median of 10 to 14 days after infection [10,11] and with RT-PCR screening data being obtained earlier in the infection.

Cq: quantification cycle; Rt: temporal reproduction number; SARS-CoV-2: severe acute respiratory syndrome coronavirus 2.

Panels A–D show the the 7-day rolling averages of the time series of the ratio of positive tests (in purple), the median (in green) and skewness (in cyan) of the daily Cq residual distribution and *Rt* (in black). Panels E–F show the error made by a prediction using only *Rt* data (red dots) and the potential improvement made by including exogeneous data.

To further assess the usefulness of Cq data, we used ARIMA models to predict *Rt* dynamics over 7 days. We compared models without any exogenous data to models that also included exogenous time series (either median or skewness of estimated Cq values distribution, or the fraction of positive tests [1]). As expected, the prediction error made using only endogenous data (*Rt*) was low in periods where *Rt* variations were limited. Furthermore, we found that adding exogenous data improved the prediction, especially when strong shifts in *Rt* were occurring (Figure 2). The Cq values tended to provide a better reduction in the error of the prediction than the ratio of positive tests.

## Discussion

This analysis of a large database of RT-PCR tests performed in all of the French regions during the first two COVID-19 pandemic waves in 2020 confirms that population-level Cq values are noisy since even a linear model that featured 91 degrees of freedom did not explain the majority of the variance. However, owing to the law of large numbers, we detected several effects that are in line with biological observations and with virological properties. For instance, our finding that Cq values decreased as a function of the number of days after symptoms onset is consistent with longitudinal follow-up [3]. Another study also reported lower Cq values when the test was performed in symptomatic individuals [12]. The same study found that men had slightly lower Cq values than women, which was not significant in our analysis. Similarly, the difference we detected depending on the virus gene targeted by the RT-PCR assay used can be interpreted in the light of known differences in mRNA copy numbers between genes depending on their distance from the 3’ end [13]. We also found slightly higher Cq values in samples collected from nasopharyngeal swabs compared with other samples (mainly lower respiratory tracts), but this is probably because the latter tests were performed in patients with more severe symptoms. Regarding the link we found between age and Cq values, although there are some mechanistic hypotheses as to why virus load would increase with age, such as variations in ACE2 receptor expression and immunosenescence [14], the evidence was mixed, with some studies reporting a decreasing trend [15] and others not [12,16]. Here, using a multivariate approach on a large dataset allowed us to unravel a strong and significant decrease of Cq values with age. 

Finally, we found Cq differences associated with the gene targeted by the RT-PCR assay that are consistent with the life cycle of the virus. As stressed by Michalakis et al. [4], since coronaviruses are (+)ssRNA viruses, they use the same RNA matrix for replication and transcription, both being amplified by diagnostic assays. The problem is that the RNA matrix for transmission is not the same for each gene as *Coronaviridae* transcripts can produce subgenomic mRNAs that lack part of the genome [17]. As a consequence, and as shown in cell cultures [13], genes at the 5’ end of the genome are under-represented. This is consistent with our result where assays targeting the gene at the 3’ end (the N gene) tend to have lower Cq values than assays targeting genes at the 5’ end (the ORF1 and S genes). Note that an alternative explanation could be that some probes target more conserved areas of the SARS-CoV-2 than others [18].

A limitation of our study is that although our dataset stands out by its size and its level of details, it is restricted to a single country where testing effort varied, both on a temporal and on a spatial scale (Supplementary Figure S2). Although the testing behaviour can be assumed to be homogeneous, epidemic spread was different between regions, which could blur the relationship between Cq values and *Rt* at the national level. Performing similar analyses in other European countries and regions can also be particularly informative. In our study, we chose to analyse all the tests performed that had a Cq value. This is debatable since high Cq values can be due to noise and this is the point of implementing cut-offs. However, Cq values are known to increase during the course of an infection [3] and these high values could correspond to patients detected in a late stage, which is expected to be more frequent in a declining epidemic [9]. To control for this potential bias, we also performed the analysis on a dataset without the tests with a 'negative' result. Finally, this analysis was conducted at the end of the year 2020 but since then, as in most countries, the emergence of variants has altered epidemiological dynamics in France [19,20] and early reports suggest that the Cq value measured could depend on the variant causing the infection [21-25]. Vaccination has also changed the picture as indicated by Cq estimations in vaccine breakthrough infections [24,25] and should be included as a host factor in future analyses.

As pointed out elsewhere, care should be taken when interpreting Cq values because of technical issues (different assays may yield higher or lower values) and biological issues (coronaviruses produce subgenomic RNAs of different lengths) [4]. However, in this analysis, we do not attempt to link Cq values to viral loads but rather analyse raw values at a population level. A promising output of this analysis is the possibility to use Cq values as an early signal to detect changes in epidemic behaviour, e.g. *Rt* values. Indeed, in 2020, our most robust descriptors of the epidemic on a short time scale originated from hospital admission data, but these still have a considerable delay relative to the status of the epidemic since patients are hospitalised 2 weeks after infection [10,11]. The ratio of positive tests performed in the population of interest can, in theory, provide earlier insights but it suffers from strong sampling biases. We show that accounting for population-based variations in Cq values can improve *Rt* predictions on a 7-day period. This is consistent with a recent study which found a correlation between *Rt* and Cq distribution skewness using data from nasopharyngeal specimens collected from staff and residents in four long-term care facilities in Massachusetts, United States [9]. Note that, contrarily to us, this earlier study did not factor in individual data such as patient age or symptomatic status, and it did not perform a cross-validation analysis that would control for temporal autocorrelation issues.

Our results show that analysing a large dataset of Cq values from screening tests can filter out the important amount of noise in these values. Inclusion of Cq values in routine surveillance calls for an adaptation to the current state of the epidemic, especially the evolution of variants and the increase in vaccination coverage, but also the integration with other types of data such as mobility data [26-28]. 

## Conclusion

In many European Union countries, the qualitative outcome of SARS-CoV-2 screening tests are already aggregated in national databases to monitor epidemic spread. Adding Cq values as well as basic metadata (such as the RT-PCR assay used or, to a lesser extent, the age and sex) could be done while there should be minimal economical and ethical challenges. Our results call for a better integration of Cq values in national and European surveillance programmes to monitor epidemics caused by SARS-CoV-2 or other human viruses, especially since these data raise fewer ethical concerns than other sources such as mobility data.

## Supplementary Data

899000 Supplement
