# Supplementary material for: Epidemiological and clinical insights from SARS-CoV-2 RT-PCR crossing threshold values, France, January to November 2020
Source: Euro Surveill. 2022 Feb 10;27(6):2100406. doi: 10.2807/1560-7917.ES.2022.27.6.2100406 (PMC8832522; doi:10.2807/1560-7917.ES.2022.27.6.2100406)
Supplement: Supplement [file 21-00406_ALIZON_Supplement.pdf]

This supplementary material is hosted by Eurosurveillance as supporting information alongside the *Epidemiological and clinical insights from SARS-CoV-2 RT-PCR crossing threshold values* on behalf of the authors who remain responsible for the accuracy and appropriateness of the content. The same standards for ethics, copyright, attributions and permissions as for the article apply. Supplements are not edited by *Eurosurveillance* and the journal is not responsible for the maintenance of any links or email addresses provided therein.

Supplementary materials

Contents

S1 Authors contributions 1

S2 Data and scripts 1

S3 SFM COVID-19 study group 1

S4 Supplementary Methods 3

S4.1 Initial data filtering . . . 3

S4.2 Temporal reproduction number (R_t) . . . 3

S4.3 Statistical analyses . . . 3

S4.3.1 Linear model . . . 3

S4.3.2 Time series analyses . . . 4

S4.3.3 Predictive analyses . . . 4

S4.3.4 Figure 1 C_q values . . . 5

S5 Supplementary Figures 6

S6 Supplementary Tables 9

S1 Participating laboratories

The 21 virology laboratories who contributed data to this study are from CERBA, BIOGROUP, CHU La Pitié Salpêtrière APHP, CNR IPP, CHU de Bordeaux, CHU de Strasbourg, CHU de Rennes, CHU de Bordeaux, CHU de Saint-Etienne, CHU de Nimes, CHU HEGP APHP, IHU Méditerranée Infection, CHU de Nantes, CHU de Nancy, CHU de Dijon, CHU d'Angers, CHU de Limoges, CHU de Caen, CHU de Montpellier, CHU Saint Louis APHP, CHU de Clermont-Ferrand, CHU Henri Mondor, CHU Bichat Claude Bernard APHP and CHU Cochin APHP.

S2 Data and scripts

The final data set analyzed along with the R scripts will be made available upon publication.

S3 SFM COVID-19 study group

The participants of the French Microbiology Society (SFM) COVID-19 study group are LINA Gérard (president of the SFM, CHU Lyon), VABRET Astrid (CHU de Caen), ADNET Justine (CHU de Caen), ROQUEBERT Benedicte (Cerba), DUCANCELLE Alexandra (CHU d'Angers), LE GUILLOU-GUILLEMETTE Hélène (CHU d'Angers), BOUTHRY Elise (CHU d'Angers), LUNEL-FABIANI Françoise (CHU d'Angers), PIVERT Adeline (CHU d'Angers), APAIRE-MARCHAIS Véronique (CHU d'Angers), ROGER Steven (CHU d'Angers), Chakib Alloui (Avicenne), Ségolène Brichler (Avicenne), Emmanuel Gordien (Avicenne), MIRAND Audrey (CHU de Clermont-Ferrand), ARCHIMBAUD Christine (CHU de Clermont-Ferrand), BREBION Amélie (CHU de Clermont-Ferrand), REGAGNON Christel (CHU de Clermont-Ferrand), CHABROLLES Hélène (CHU de Clermont-Ferrand), BISSEUX Maxime (CHU de Clermont-Ferrand), COMBES Patricia (CHU de Clermont-Ferrand), Hélène Jeulin (CHU de Nancy), Véronique Venard (CHU de Nancy), Evelyne Schvoerer (CHU de Nancy), Anne Lebouter (Henri Mondor APHP), Souraya Khouider (Henri Mondor APHP), Alexandre Soulier (Henri Mondor APHP), Aurelie Gourgeon (Henri Mondor APHP), Bellecave Pantxika (CHU de Bordeaux), Busson Laurent (CHU de Bordeaux), Garrigue Isabelle (CHU de Bordeaux), Lafon Marie-Edith (CHU de Bordeaux), Trimoulet Pascale (CHU de Bordeaux), Bruno Pozzetto (CHU de Saint-Etienne), Thomas Bourlet (CHU de Saint-Etienne), Sylvie Gonzalo (CHU de Saint-Etienne), Rémi Labetoulle (CHU de Saint-Etienne), Rogez Sylvie (CHU de Limoges), Alain Sophie (CHU de Limoges), Marianne Coste-Burel (CHU de Nantes), Virginie Ferré (CHU de Nantes), Berthe-Marie Imbert-Marcille (CHU de Nantes), Pierre Edouard Fournier (IHU Méditerranée infection), Petit Paul Rémi (IHU Méditerranée infection), Luciani Léa (IHU Méditerranée infection), Zandotti Christine (IHU Méditerranée infection), Charre Caroline (Cochin APHP), Mariaggi Alice-Andrée (Cochin APHP), Méritet Jean-François (Cochin APHP), Rozenberg Flore (Cochin APHP), Febreau Christine (CHU de Rennes), Comacle Pauline (CHU de Rennes), Lagathu Gisèle (CHU de Rennes), Maillard Anne (CHU de Rennes), Grolhier Claire (CHU de Rennes), Pronier Charlotte (CHU de Rennes), David Boutolleau (La Pitié Salpêtrière APHP), Anne-Geneviève Marcelin (La Pitié Salpêtrière APHP), Vincent Calvez (La Pitié Salpêtrière APHP), Stéphane Marot (La Pitié Salpêtrière APHP), Sepideh Akhavan (La Pitié Salpêtrière APHP), Basma Abdi (La Pitié Salpêtrière APHP), Marc Wirden (La Pitié Salpêtrière APHP), Cathia Soulié (La Pitié Salpêtrière APHP), Aude Jary (La Pitié Salpêtrière APHP), Elisa Teyssou (La Pitié Salpêtrière APHP), Sylvie van der Werf (CNR IPP), Vincent Enouf (CNR IPP), BOUDET Agathe (CHU de Nîmes), CARLES Marie-Josee (CHU de Nîmes), PERE Hélène (HEGP APHP), BELEC Laurent (HEGP APHP), IZQUIERDO Laure (HEGP APHP), RODARY Julien (HEGP APHP), BAILLARD Jean-Louis (HEGP APHP), RIBEYRE Tatiana (HEGP APHP), SALIBA Madelina (HEGP APHP), ROGER Alicia (HEGP APHP), GARNIER Nathalie (HEGP APHP), ROBILLARD Nicolas (HEGP APHP), Le Goff Jérôme (Saint Louis APHP), Delaugerre Constance (Saint Louis APHP), Chaix Marie-Laure (Saint Louis APHP), Feghoul Linda (Saint Louis APHP), Mahjoub Nadia (Saint Louis APHP), Maylin Sarah (Saint Louis APHP), Schnepf Nathalie (Saint Louis APHP), Alfaisal Jamal (Saint Louis APHP), AGNELLO Davide (CHU de Dijon), AUVRAY Christelle (CHU de Dijon), BELLIOT Gaël (CHU de Dijon), BOUR Jean-Baptiste (CHU de Dijon), CASENAZ Alice (CHU de Dijon), GUILLOTIN Florence (CHU de Dijon), MANOHA Catherine (CHU de Dijon), SI-MOHAMMED Ali (CHU de Dijon), TAN Rithy-Nicolas (CHU de Dijon), Diane Descamps (Bichat APHP), Nadhira Houhou-Fidouh (Bichat APHP), Charlotte Charpentier (Bichat APHP), Houria Ichou (Bichat APHP), Florence Damond (Bichat APHP), Quentin

Le Hingrat (Bichat APHP), Valentine Ferré (Bichat APHP), Lucile Larrouy (Bichat APHP), Vincent Mackiewicz (Bichat APHP), Gilles Collin (Bichat APHP), FAFI-KREMER Samira (CHU de Strasbourg), GALLAIS Floriane (CHU de Strasbourg), LAUGEL Elodie (CHU de Strasbourg), BENOTMANE Ilies (CHU de Strasbourg), VELAY Aurélie (CHU de Strasbourg), and WENDLING Marie-Josée (CHU de Strasbourg)

S4 Supplementary Methods

S4.1 Initial data filtering

The SFM sent a query to collect anonymous RT-PCR test results data from 19 public and 2 private laboratories. The response files were curated manually and merged using R. Test values without any C_q (also referred to as C_t) values (negative tests) were removed.

This led to an initial global data set from $2,220,212$ individuals. Removing non-numerical C_q values (usually a qualitative description of a negative result) decreased this number by 30% from $10,668,371$ to $7,516,936$ C_q values (note that most tests are usually associated with more than a single value since it can have multiple targets). We then removed all the C_q values equal to zero, which left us with $1,969,043$ values.

Finally, we performed some extra filtering to remove aberrant C_q values (greater than 100), test with missing values for sampling French department, qualitative result, or RT-PCR test used. This left us with $1,299,447$ values originating from $824,446$ individuals. Further details are available in Table S1.

For each individual, we retained only the earliest sample therefore analyzing $1,129,437$ C_q values.

S4.2 Temporal reproduction number (R_t)

The temporal reproduction number (denoted R_t) was computed on the COVID-19 hospital admission incidence time series established by the national public health agency (Santé Publique France) and accessible at this website. Because of strong daily variations (especially on week-ends), we first transformed the time series using a 7-days rolling average. We then used the EpiEstim method [1] and the eponym package in R [2].

Earlier studies have reported that, for patients who develop severe symptoms, the median time between infection and hospital admission is in the order of 14 days [3, 4].

S4.3 Statistical analyses

All the analyses were performed in R version 3.6.3.

S4.3.1 Linear model

The linear model was performed in individuals from 1 to 90 years old. We also removed C_q values associated to internal controls because they caused the distribution of the residuals to be non-Gaussian. Quantitative factors, namely $R(t)$, date, and age were scaled and centered using the `scale()` base function in R.

One scaled age unit corresponded to 20.08 years and one scaled date unit corresponded to 71.49 days.

The model was formulated as follows in R:

```
modele_general = lm(Ct ~ Rt*date + age + sexe + target_gene +
assay_PCR + id_lab  + symptoms + result +
sample_type + sampling_facility + control_variable, data)
```

The adjusted R^2 of the model was 38.8% and the distribution of the residuals Gaussian (Figure S3A).

The model was analyzed using an ANOVA assuming type-II errors because of the unbalanced nature of the data set. All the variable were found to be extremely significant (p-value $< 10^{-6}$), except for R_t (p-value of 0.68) the control variable (p-value of 0.0131), sampling facility (p-value of 0.0135), sex (p-value of 0.0021), interaction between R_t and date (p-value of 0.000842).

S4.3.2 Time series analyses

To analyze the time series of reproduction number and C_q values we restricted the data to tests performed after July 1, 2021, in a screening context, using nasopharyngeal swabs, in individuals aged from 6 to 80. These assumptions were made such that C_q values would reflect the state of the ongoing epidemic. Finally, we excluded values from internal control genes. Overall, we analysed 234,782 C_q values from 110,227 individuals.

To correct for other potential confounding factors, we first performed a linear model.

The analyses were performed on the residuals of the linear model. For each day, we computed the median and the skewness of the distribution. Skewness was computed using the formula $\frac{n}{(n-1)(n-2)} \sum_i \left(\frac{x_i - \text{mean}(x_i)}{\text{Var}(x_i)} \right)^3$, with x_i the individual values and n the total number of values. We then computed a 7-day rolling mean to buffer daily variations.

The temporal reproduction number was computed as indicated above. We also analyzed a 7-day rolling mean.

Cross-correlation function analyses were performed using the ccf function in R.

S4.3.3 Predictive analyses

We used ARIMA models (implemented in the R package forecast) to predict the hospital-admission temporal reproduction number (R_t) from past observations.

For each date, predictions were evaluated in terms of the mean absolute percentage error (MAPE) for horizons of 7 days in the future based on coefficients learned from past data starting on July 29th, 2020. More precisely, for each date we compared the temporal reproduction number data $\{D_k; k = 1, \ldots, 7\}$ with the seven-day model forecast $\{F_k; k = 1, \ldots, 7\}$ by

$$\text{MAPE} = \frac{1}{7} \sum_{k=1}^{7} \left| \frac{D_k - F_k}{D_k} \right|$$

We considered 4 types of data:

1. past R_t data (i.e. endogenous data),
2. quartiles from C_q residuals (to remove biases),
3. skewness from Ct residuals,

4. national positive test ratio collected from https://covid.ourworldindata.org/data/.

The residuals of C_q were obtained from the following linear model:

```
lm(Ct ~ age + target_gene*assay_PCR + id_lab, data)
```

Our goal was to see if adding exogenous data, i.e. C_q values and proportion of positive tests, increased prediction precision.

Models were tuned with the `auto.arima` function, and untuned models were run with $p = 9$, $d = 2$, and $q = 0$ as default parameters, based on the cross-correlation analysis between R_t and C_q time series (Figure S3).

Prediction improvement by models using in addition exogenous data relative to model using past values of R_t only was defined by

$$\Delta\text{MAPE} = \text{MAPE}_{\text{Rt}} - \text{MAPE}_{\text{Rt+exo}}$$

S4.3.4 Figure 1 C_q values

To visualize the effect of the different factors, we first perform a linear model to correct for the effect of confounding factors.

We used the general model described above but removed the effect 'control_variable'. For panels E and F, we also scaled the age and the R_t values.

The C_q value itself was generated using the `fitted.values` function in R.

References

[1] Cori A, Ferguson NM, Fraser C, Cauchemez S. A New Framework and Software to Estimate Time-Varying Reproduction Numbers During Epidemics. Am J Epidemiol. 2013 Nov;178(9):1505–1512.

[2] Thompson RN, Stockwin JE, van Gaalen RD, Polonsky JA, Kamvar ZN, Demarsh PA, et al. Improved inference of time-varying reproduction numbers during infectious disease outbreaks. Epidemics. 2019;29:100356.

[3] Salje H, Kiem CT, Lefrancq N, Courtejoie N, Bosetti P, Paireau J, et al. Estimating the burden of SARS-CoV-2 in France. Science. 2020;369(6500):208–211.

[4] Sofonea MT, Reyné B, Elie B, Djidjou-Demasse R, Selinger C, Michalakis Y, et al. Memory is key in capturing COVID-19 epidemiological dynamics. Epidemics. 2021;35:100459.

S5 Supplementary Figures

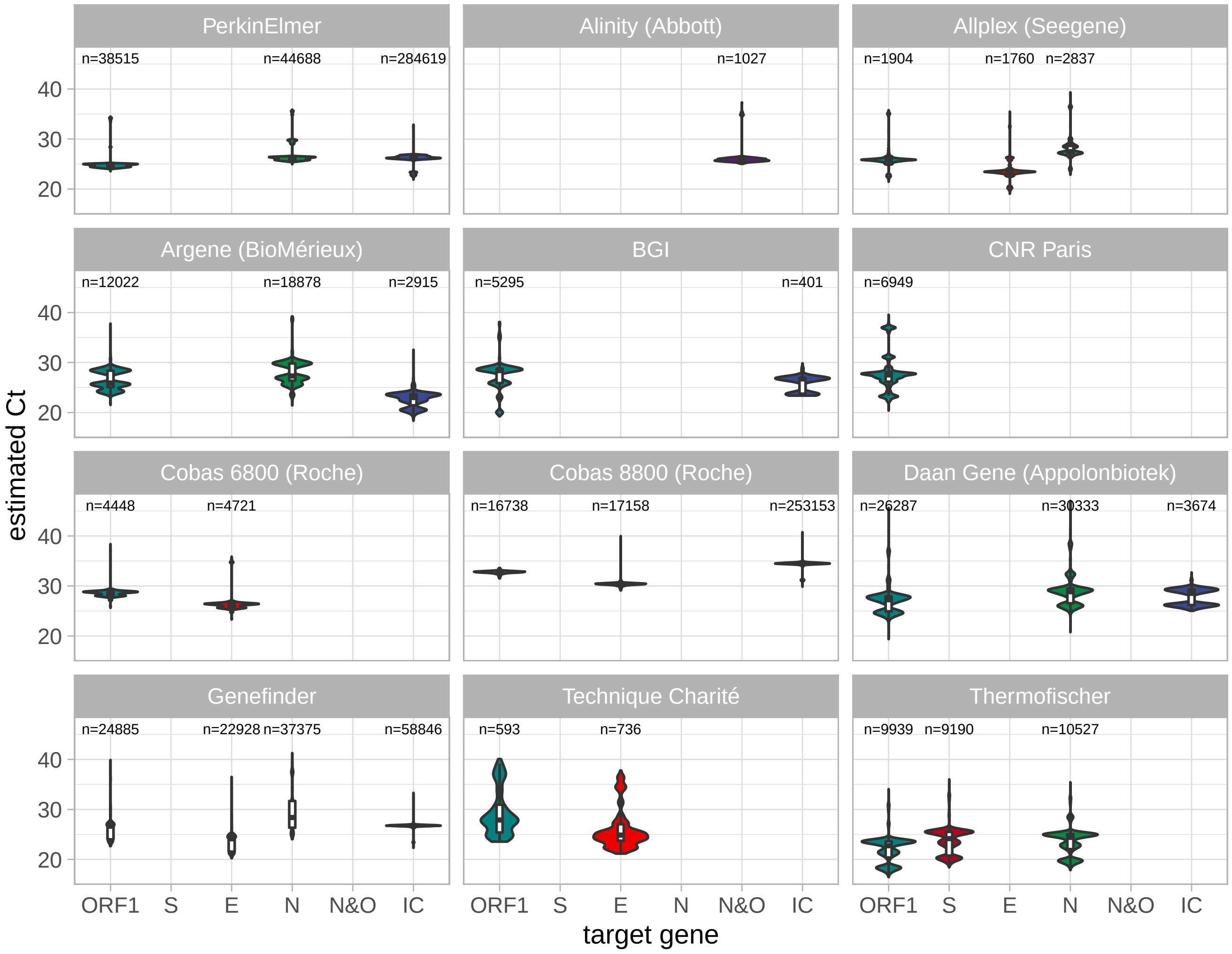

Figure S1: **Effect of the RT-PCR assay used on the estimated C_q value as a function of the targeted genomic area.** Only tests with at least 1,000 C_q values are shown. IC stands for 'internal control'.

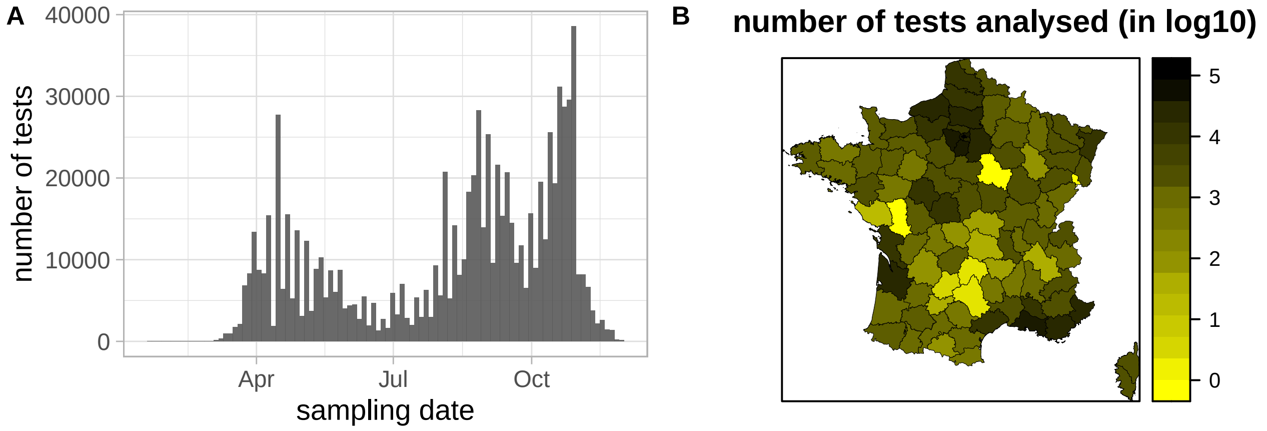

Figure S2: **Number of tests performed per day (A) and per French department (B).**

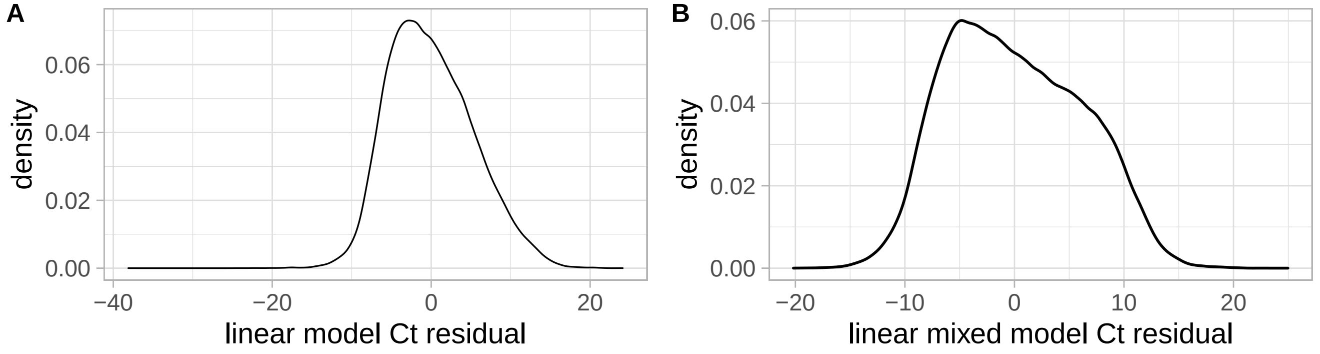

Figure S3: **Distribution of the C_q residuals value.** A) For the linear model used for the main analysis shown in Table S2, B) for the linear model used to generate residuals for the R_t time series analysis.

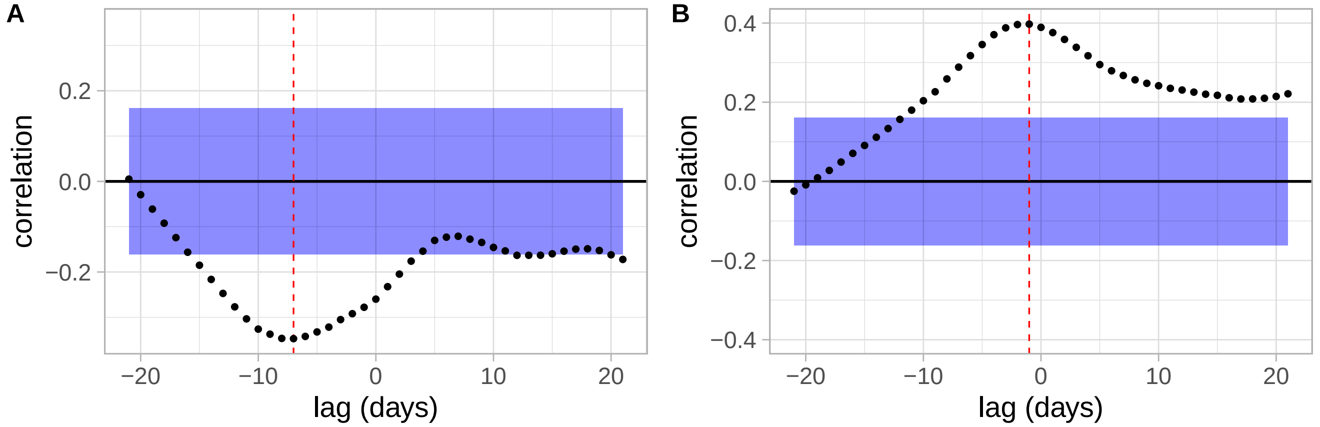

Figure S4: **Cross correlation functions between R_t and A) the median or B) the skewness of the C_q residuals distribution.** The blue shaded areas show the non-significant values (with a 95% threshold) and the red vertical dotted lines the lag with the highest significant correlation. Note that the lag is smaller for the skewness than for the median of the distribution.

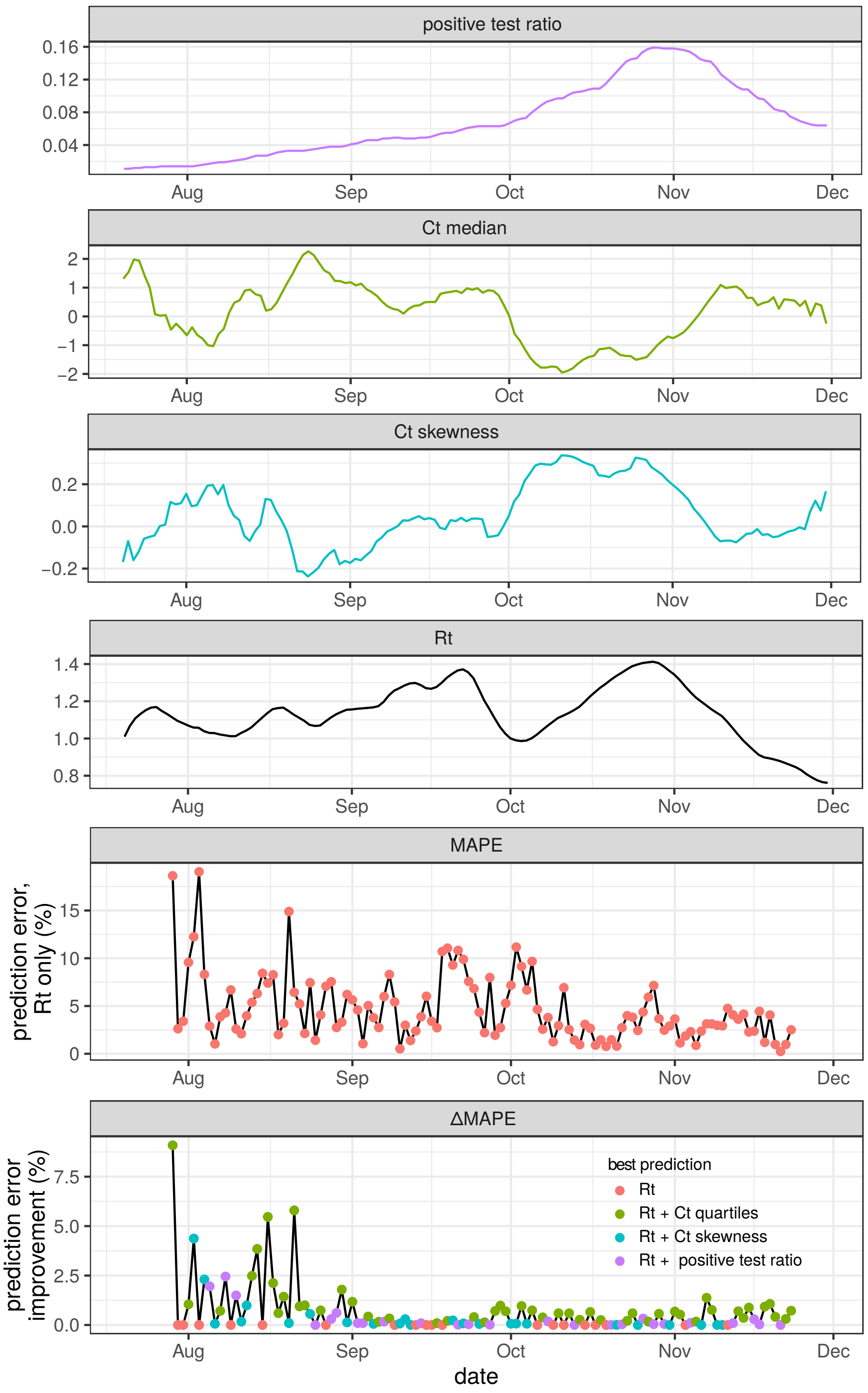

Figure S5: **Predicting temporal reproduction number (R_t) from time time series with untuned ARIMA parameters** $p = 9, d = 2, q = 0$ The top four panels show the the 7-days rolling averages of the time series of the ratio of positive tests (in purple), the median (in green) and skewness (in cyan) of the daily C_q residuals distribution, and R_t (in black). The bottom panels show the error made by a prediction using only R_t data (red dots) and the potential improvement made by including exogeneous data.

S6 Supplementary Tables

Table S1: **Description of the dataset variables.** For real numbers, we show the median and the 95% confidence interval. For categorical variables, we either indicate the number of factors or the number of occurrences n for each factor.

Variable	Description	Details	Values
department	French administrative department where the sampling was performed	categorical	97 departments
id_lab	laboratory associated with the sampling	categorical	128 labs
control_variable	a control variable created using the last digit of the patient anonymity number	categorical	10 values
id_patient	participant anonymity number	categorical	$825,446$ ids
date	sampling date	date	min = 01/21/2020<br>max = 30/11/2020
sampling_facility	type of facility where the sampling was performed	*city screening*<br>*aged care home*<br>*hospital*<br>*prison*<br>*missing*	$n = 1,008,307$<br>$n = 3,822$<br>$n = 72,682$<br>$n = 45$<br>$n = 44,570$
sample_type	clinical localization sampled	*nasopharyngeal*<br>*other*	$n = 1,086,004$<br>$n = 35,660$
assay_PCR	the description of the RT-PCR assay used	categorical	13 assays
target_gene	SARS-CoV-2 genomic area corresponding to the C_q value	*N*<br>*E*<br>*S*<br>*ORF1*<br>*N & ORF1*<br>*internal control*	$n = 199,256$<br>$n = 55,717$<br>$n = 9,953$<br>$n = 227,192$<br>$n = 1,196$<br>$n = 636,123$
C_q	amplification cycles required to reach the signal (0 values are removed)	real	28.10 [18.30; 37.37]
R_t	temporal reproduction number (see Supplementary methods for details)	real	1.15 [0.76; 1.41]
result	qualitative result of the test	*positive*<br>*negative*<br>*weak positive*	$n = 524,065$<br>$n = 585,293$<br>$n = 20,079$
age	participant age (in years)	integer	48 [25; 83]
sex	patient sex	*female*<br>*male*	$n = 637,212$<br>$n = 492,225$
symptoms	number of days between symptoms onset and testing	*less than 4*<br>*4 to 7*<br>*8 to 12*<br>*more than 12*<br>*missing*	$n = 107,417$<br>$n = 22,308$<br>$n = 6,070$<br>$n = 1,945$<br>$n = 991,697$

Table S2: **Linear model detailed results.** Estimates reflect differences in C_q. For qualitative factors, the reference value is indicated. Note that some control variables are found to be significant according to a classical 5% significance threshold, which indicates the need for stringer criteria for p-value significance.

Factor	Estimate	Std. Error	t value	Pr(>|t|)
(Intercept)	19.136	3.186	6.007	0
Reproduction number (scaled, Rt)	2.101	0.625	3.359	0.001
Sampling date (scaled)	-0.797	0.054	-14.71	0
Age (scaled)	-0.541	0.022	-24.25	0
Sex M (reference = F)	-0.115	0.037	-3.081	0.002
Target gene S (reference = N)	1.189	0.123	9.658	0
Target gene E (reference = N)	0.039	0.066	0.59	0.555
Target gene O (reference = N)	1.034	0.043	24.007	0
PCR Assay Argene (reference = Perkin)	4.641	3.061	1.516	0.129
PCR Assay BGI (reference = Perkin)	5.159	3.067	1.682	0.093
PCR Assay CNR Paris (reference = Perkin)	3.961	3.077	1.287	0.198
PCR Assay Cobas 6800 (reference = Perkin)	6.935	3.155	2.198	0.028
PCR Assay Daan Gene (reference = Perkin)	5.782	3.092	1.87	0.062
PCR Assay Genefinder (reference = Perkin)	12.147	0.92	13.204	0
PCR Assay Technique Drosten (reference = Perkin)	2.424	3.197	0.758	0.448
PCR Assay Thermofischer (reference = Perkin)	6.779	3.079	2.202	0.028
PCR Assay other (reference = Perkin)	0.433	2.846	0.152	0.879
Laboratory LAB_10	0.488	0.839	0.581	0.561
Laboratory LAB_100	0.025	0.901	0.028	0.978
Laboratory LAB_101	0.07	1.239	0.057	0.955
Laboratory LAB_102	0.744	0.96	0.774	0.439
Laboratory LAB_103	1.257	1.195	1.051	0.293
Laboratory LAB_104	-1.817	1.518	-1.197	0.231
Laboratory LAB_105	1.54	1.047	1.471	0.141
Laboratory LAB_106	0.988	0.924	1.069	0.285
Laboratory LAB_11	0.179	0.862	0.207	0.836
Laboratory LAB_110	-3.805	2.548	-1.493	0.135
Laboratory LAB_111	0.204	2.002	0.102	0.919
Laboratory LAB_112	2.113	1.519	1.392	0.164
Laboratory LAB_113	0.411	1.996	0.206	0.837
Laboratory LAB_119	4.423	3.193	1.385	0.166
Laboratory LAB_12	-1.013	0.861	-1.177	0.239
Laboratory LAB_120	5.98	3.881	1.541	0.123
Laboratory LAB_122	5.418	0.833	6.503	0
Laboratory LAB_127	0.876	0.954	0.918	0.359
Laboratory LAB_128	2.512	0.879	2.859	0.004
Laboratory LAB_13	-0.118	0.878	-0.134	0.893
Laboratory LAB_14	0.38	0.836	0.454	0.65
Laboratory LAB_15	0.734	0.839	0.874	0.382
Laboratory LAB_16	0.377	0.851	0.442	0.658
Laboratory LAB_17	1.123	0.94	1.195	0.232
Laboratory LAB_18	-0.281	0.844	-0.333	0.739
Laboratory LAB_19	-0.452	0.851	-0.531	0.595
Laboratory LAB_2	0.341	0.847	0.403	0.687
Laboratory LAB_20	0.398	0.837	0.475	0.635
(...)				

Factor	Estimate	Std. Error	t value	Pr(>|t|)
Laboratory LAB_21	-0.568	0.858	-0.663	0.507
Laboratory LAB_22	-2.641	1.064	-2.482	0.013
Laboratory LAB_23	-0.549	0.856	-0.641	0.521
Laboratory LAB_24	-0.123	0.867	-0.141	0.887
Laboratory LAB_25	-3.113	2.804	-1.11	0.267
Laboratory LAB_26	0.484	0.845	0.573	0.567
Laboratory LAB_27	-1.429	2.798	-0.511	0.609
Laboratory LAB_28	-4.177	1.338	-3.121	0.002
Laboratory LAB_29	5.144	2.798	1.839	0.066
Laboratory LAB_3	-0.084	0.842	-0.099	0.921
Laboratory LAB_30	-0.31	0.869	-0.357	0.721
Laboratory LAB_31	-0.872	0.886	-0.984	0.325
Laboratory LAB_32	1.33	0.865	1.537	0.124
Laboratory LAB_33	-0.94	1.01	-0.931	0.352
Laboratory LAB_4	0.202	0.847	0.238	0.812
Laboratory LAB_5	0.123	0.854	0.145	0.885
Laboratory LAB_6	0.081	0.85	0.096	0.924
Laboratory LAB_7	-0.159	0.838	-0.19	0.849
Laboratory LAB_77	-0.151	0.866	-0.174	0.862
Laboratory LAB_8	0.186	0.882	0.211	0.833
Laboratory LAB_83	1.262	1.099	1.148	0.251
Laboratory LAB_89	-7.817	3.051	-2.562	0.01
Laboratory LAB_9	-0.383	0.856	-0.447	0.655
Laboratory LAB_90	-7.785	3.05	-2.552	0.011
Laboratory LAB_91	-9.078	3.054	-2.972	0.003
Laboratory LAB_92	2.317	1.292	1.794	0.073
Laboratory LAB_96	-4.805	0.974	-4.934	0
Laboratory LAB_97	-2.75	2.363	-1.164	0.245
Laboratory LAB_99	-1.418	2.815	-0.504	0.615
Symptoms 4 to 7 days (reference = less than 4 days)	2.762	0.051	53.656	0
Symptoms 8 to 14 days (reference = less than 4 days)	4.901	0.089	54.92	0
Symptoms more than 14 days (reference = less than 4 days)	5.726	0.153	37.373	0
Sample type OP (reference = NP)	-1.814	0.343	-5.293	0
Sampling facility nursing home (reference = screening)	0.799	0.351	2.278	0.023
Sampling facility hospital (reference = screening)	-0.006	0.199	-0.031	0.975
Sampling facility unknown (reference = screening)	0.176	0.095	1.846	0.065
Qualitative result negative (reference = positive)	16.91	0.135	125.408	0
Qualitative result weak positive (reference = positive)	11.263	0.1	112.215	0
Control variable 1 (reference = 0)	-0.198	0.083	-2.385	0.017
Control variable 2 (reference = 0)	-0.079	0.083	-0.947	0.344
Control variable 3 (reference = 0)	-0.172	0.083	-2.071	0.038
Control variable 4 (reference = 0)	-0.143	0.083	-1.723	0.085
Control variable 5 (reference = 0)	-0.053	0.083	-0.644	0.519
Control variable 6 (reference = 0)	0.099	0.083	1.187	0.235
Control variable 7 (reference = 0)	-0.153	0.083	-1.844	0.065
Control variable 8 (reference = 0)	-0.064	0.083	-0.767	0.443
Control variable 9 (reference = 0)	-0.126	0.083	-1.527	0.127
interaction : Rt (scaled) * sampling date (scaled)	1.02	0.305	3.339	0.001

Table S3: **Linear model exlcuding negative tests from the data.** See Table S2 for additional details.

Factor	Estimate	Std. Error	t value	Pr(>|t|)
(Intercept)	27,463	3,632	7,562	0
Reproduction number (scaled, Rt)	2,085	0,633	3,292	0,001
Sampling date (scaled)	-0,839	0,055	-15,155	0
Age (scaled)	-0,543	0,023	-23,877	0
Sex M (reference = F)	-0,122	0,038	-3,232	0,001
Target gene S (reference = N)	1,206	0,124	9,739	0
Target gene E (reference = N)	0,11	0,067	1,648	0,099
Target gene O (reference = N)	1,068	0,044	24,452	0
PCR Assay Argene (reference = Perkin)	-3,547	3,513	-1,01	0,313
PCR Assay BGI (reference = Perkin)	-3,064	3,518	-0,871	0,384
PCR Assay CNR Paris (reference = Perkin)	-4,336	3,527	-1,229	0,219
PCR Assay Cobas 6800 (reference = Perkin)	-1,303	3,596	-0,362	0,717
PCR Assay Daan Gene (reference = Perkin)	-2,351	3,54	-0,664	0,507
PCR Assay Genefinder (reference = Perkin)	8,014	1,149	6,976	0
PCR Assay Technique Drosten (reference = Perkin)	-5,833	3,643	-1,601	0,109
PCR Assay Thermofischer (reference = Perkin)	-1,396	3,528	-0,396	0,692
PCR Assay other (reference = Perkin)	-7,798	3,325	-2,345	0,019
Laboratory LAB_10	0,344	0,886	0,388	0,698
Laboratory LAB_100	-0,1	0,939	-0,106	0,915
Laboratory LAB_101	-0,05	1,269	-0,039	0,969
Laboratory LAB_102	0,633	0,997	0,635	0,525
Laboratory LAB_103	1,127	1,227	0,918	0,358
Laboratory LAB_104	-1,931	1,546	-1,249	0,212
Laboratory LAB_105	1,426	1,081	1,319	0,187
Laboratory LAB_106	0,88	0,961	0,916	0,36
Laboratory LAB_11	-0,015	0,909	-0,016	0,987
Laboratory LAB_110	-3,886	2,574	-1,51	0,131
Laboratory LAB_111	0,15	2,027	0,074	0,941
Laboratory LAB_112	1,952	1,546	1,263	0,207
Laboratory LAB_113	0,347	2,022	0,171	0,864
Laboratory LAB_119	-3,83	3,637	-1,053	0,292
Laboratory LAB_12	-1,228	0,907	-1,353	0,176
Laboratory LAB_120	5,828	3,91	1,491	0,136
Laboratory LAB_122	5,247	0,88	5,964	0
Laboratory LAB_127	0,844	0,989	0,854	0,393
Laboratory LAB_128	2,374	0,918	2,586	0,01
Laboratory LAB_13	-0,327	0,924	-0,354	0,723
Laboratory LAB_14	0,202	0,883	0,229	0,819
Laboratory LAB_15	0,591	0,886	0,667	0,505
Laboratory LAB_16	0,273	0,898	0,304	0,761
Laboratory LAB_17	0,915	0,983	0,931	0,352
Laboratory LAB_18	-0,463	0,891	-0,519	0,604
Laboratory LAB_19	-0,657	0,898	-0,732	0,464
Laboratory LAB_2	0,187	0,893	0,209	0,834
Laboratory LAB_20	0,215	0,884	0,243	0,808
(...)				

Factor	Estimate	Std. Error	t value	Pr(>|t|)
Laboratory LAB_21	-0,795	0,904	-0,879	0,379
Laboratory LAB_22	-2,952	1,104	-2,675	0,007
Laboratory LAB_23	-0,726	0,902	-0,804	0,421
Laboratory LAB_24	-0,302	0,913	-0,331	0,741
Laboratory LAB_25	-3,557	2,833	-1,256	0,209
Laboratory LAB_26	0,322	0,892	0,361	0,718
Laboratory LAB_27	-1,649	2,826	-0,583	0,56
Laboratory LAB_28	-4,389	1,373	-3,198	0,001
Laboratory LAB_29	4,772	2,826	1,689	0,091
Laboratory LAB_3	-0,25	0,889	-0,281	0,779
Laboratory LAB_30	-0,53	0,915	-0,579	0,563
Laboratory LAB_31	-1,067	0,932	-1,145	0,252
Laboratory LAB_32	1,15	0,911	1,263	0,207
Laboratory LAB_33	-1,111	1,056	-1,051	0,293
Laboratory LAB_4	0,038	0,894	0,043	0,966
Laboratory LAB_5	-0,072	0,901	-0,08	0,936
Laboratory LAB_6	-0,114	0,897	-0,127	0,899
Laboratory LAB_7	-0,335	0,885	-0,378	0,706
Laboratory LAB_77	-0,321	0,912	-0,352	0,725
Laboratory LAB_8	-0,019	0,928	-0,021	0,983
Laboratory LAB_83	1,063	1,141	0,932	0,352
Laboratory LAB_89	-12,026	3,447	-3,488	0
Laboratory LAB_9	-0,556	0,903	-0,616	0,538
Laboratory LAB_90	-12,037	3,447	-3,492	0
Laboratory LAB_91	-13,357	3,451	-3,871	0
Laboratory LAB_92	2,199	1,322	1,663	0,096
Laboratory LAB_96	-4,926	1,013	-4,864	0
Laboratory LAB_97	-2,859	2,388	-1,197	0,231
Laboratory LAB_99	-1,576	2,841	-0,555	0,579
Symptoms 4 to 7 days (reference = less than 4 days)	2,824	0,052	54,01	0
Symptoms 8 to 14 days (reference = less than 4 days)	5,044	0,091	55,346	0
Symptoms more than 14 days (reference = less than 4 days)	6,068	0,159	38,273	0
Sample type OP (reference = NP)	-1,837	0,348	-5,277	0
Sampling facility nursing home (reference = screening)	0,769	0,354	2,172	0,03
Sampling facility hospital (reference = screening)	-0,039	0,201	-0,196	0,845
Sampling facility unknown (reference = screening)	0,165	0,096	1,713	0,087
Qualitative result weak positive (reference = positive)	11,229	0,101	110,918	0
Control variable 1 (reference = 0)	-0,2	0,084	-2,37	0,018
Control variable 2 (reference = 0)	-0,076	0,084	-0,897	0,37
Control variable 3 (reference = 0)	-0,186	0,084	-2,206	0,027
Control variable 4 (reference = 0)	-0,143	0,085	-1,688	0,091
Control variable 5 (reference = 0)	-0,063	0,084	-0,745	0,456
Control variable 6 (reference = 0)	0,105	0,085	1,237	0,216
Control variable 7 (reference = 0)	-0,162	0,084	-1,924	0,054
Control variable 8 (reference = 0)	-0,066	0,085	-0,784	0,433
Control variable 9 (reference = 0)	-0,13	0,084	-1,546	0,122
interaction : Rt (scaled) * sampling date (scaled)	1,016	0,309	3,285	0,001
